# Trends in rates and causes of hospitalization among people living with HIV in the antiretroviral therapy era: A retrospective cohort study in China, 2008–2020

**DOI:** 10.3389/fpubh.2022.1000942

**Published:** 2022-11-08

**Authors:** Ying Liu, Yiwei Hao, Jiang Xiao, Liang Wu, Hongyuan Liang, Junyan Han, Hongxin Zhao

**Affiliations:** ^1^National Center for Infectious Diseases, Clinical and Research Center of Infectious Diseases, Beijing Ditan Hospital, Capital Medical University, Beijing, China; ^2^Department of Medical Records and Statistics, Beijing Ditan Hospital, Capital Medical University, Beijing, China; ^3^Biomedical Innovation Center, Beijing Shijitan Hospital, Capital Medical University, Beijing, China

**Keywords:** HIV, hospitalization, antiretroviral therapy, AIDS-defining events, non-AIDS-defining events

## Abstract

**Background:**

The introduction of antiretroviral therapy (ART) has resulted in marked reductions in morbidity among people living with HIV (PLWH). Monitoring the hospitalizations of PLWH is important in evaluating the quality of healthcare and forecasting the co-morbidity pattern. We aimed to describe the trends in the rates and causes of hospitalization among PLWH who initiated ART in an HIV-designated hospital in China.

**Methods:**

PLWH who initiated ART and were hospitalized in Beijing Ditan Hospital from 2008 to 2020 were selected for the study. Hospitalizations were classified based on AIDS-defining events (ADEs), non-AIDS-defining events (nADEs), and other causes. Hospitalization rates were calculated in terms of person-years, with risk factors determined by Poisson regression. The proportion of hospitalization causes at different ART treatment statuses was also evaluated.

**Results:**

A total of 9,404 patients (94.7% were male patients) were included, contributing to 49,419 person-years. Overall, 1,551 PLWH were hospitalized for 2,667 hospitalization events, among which 60.4% of hospitalizations were due to ADEs, 11.4% were due to nADEs, and 28.2% were due to other causes. Unadjusted hospitalization rates decreased for all causes and all three diagnostic categories with year. After adjusting for the variables that changed substantially over time, ADE-related [IRR, 1.01 (0.96–1.05)] and nADE-related hospitalization rates [IRR, 0.92 (0.84–1.01)] appeared stable. Hospitalization for ADEs constituted an increasing proportion over time (36.3% in 2008–57.4% in 2020), especially in ART-naive inpatients (43.8% in 2008–83.3% in 2020). The proportion of nADE-related hospitalizations remained low (9.0% in 2008–15.4% in 2020). Hospitalization rate was highest for patients treated with ART during the first 6 months after ART initiation (46.2%) when ADEs were still the leading cause of hospitalizations (30.6%). Older age, non-men who have sex with men transmission, late presenters, HIV viral load (VL) > 50 copies/mL, and CD4 counts ≤ 200 cells/μL were associated with a higher hospitalization risk (all *P* < 0.05).

**Conclusion:**

Despite some progress, ADEs remain the most common and serious problem among PLWH in China. In order to avoid deteriorating to the stage of needing hospitalization, more work is needed to diagnose and treat HIV infection earlier.

## Introduction

The global expansion of access to antiretroviral therapy (ART) has led to a substantial decline in HIV-related morbidity and mortality. In China, the introduction of the National Free ART Program in 2003 by the National Center for AIDS/STD Control and Prevention has dramatically increased the ART coverage among people living with HIV (PLWH) (1). However, the number of newly diagnosed cases and HIV-related deaths has been increasing (2). Monitoring the hospitalizations of PLWH plays an important role in evaluating the quality of healthcare assistance, especially the effectiveness of ART, and in forecasting the co-morbidity pattern (3–5). A declining rate of hospitalizations has been reported in high-income countries, particularly for AIDS-defining events (ADEs) (6–8). Hospitalization rates for non-AIDS-defining events (nADEs) have been declining slowly or remaining stable, with a few studies reporting increasing hospitalization rates due to cancer, cardiovascular disease, and renal disease (9–12). Yet, in China, studying hospitalizations of PLWH has been challenging. Also, data remain scarce on the distribution and determinants of HIV-related hospitalizations over time.

In this study, we examined hospitalization trends and causes among PLWH as a proxy for severe complications over 13 years in the Capital Medical University Affiliated to Beijing Ditan Hospital, an HIV-designated hospital providing medical services to patients from local regions (Beijing, Tianjin and surrounding Hebei province) as well as those referred from other provinces and municipalities of China. We also assessed the impact of demographic and clinical characteristics on the risk of hospitalizations.

## Methods

### Data source

To identify the population of PLWH hospitalized in Capital Medical University Affiliated Beijing Ditan Hospital, we performed a deterministic matching between the China Disease Prevention and Control Information System (CDPCIS) and Tongren Medical Information System. The former contained information about clinic visits and follow-ups on all PLWH diagnosed with HIV/AIDS since 2004, and the latter, which was established in 2008, included medical record data for all hospitalizations in Beijing Ditan Hospital. HIV admissions were identified based on the International Classification of Diseases, Tenth Revision, Clinical Modification (ICD-10) HIV diagnosis code. Data collection was approved by the Human Science Ethical Committee of Beijing Ditan Hospital, Capital Medical University (No. 2021-22-01).

### Study participants

Eligibility criteria for the matching process are as follows: patients ≥ 18 years who started ART in Beijing Ditan Hospital and those who had at least one CD4 count performed or admitted to the inpatient, outpatient, or emergency room in a calendar year until they were transferred to other medical institutions or died. Person-time was censored at lost to follow-up (LTFU), defined as 12 months with no clinic or laboratory visit, but patients contributed additional person-time if they reentered HIV care.

### Matching process

Hospitalizations with same-day discharge were excluded from the matching process because these could not be distinguished from outpatient procedures (e.g., the cerebrospinal fluid reexaminations for neurosyphilis, perianal abscess, and mild trauma). The data from Tongren Medical Information System were de-duplicated at the individual level using a unique identifier. A total of four identifying elements including full name, birthday, sex, and patient residential ZIP code were matched between the CDPCIS and Tongren Medical Information System. Patient records matching these four identifying elements were assessed as “full match records” (*N* = 1,479), and those matching one to three identifying elements were assessed as “partial match records” (*N* = 243). No matched identifying elements were assessed as “no match records” and were removed from both input data sets (*N* = 4,539). The partial match records were identified by manual review.

### Analytic variables

Demographic variables and HIV-related characteristics were obtained from the CDPCIS. Characteristics and outcomes of hospitalizations were obtained from the Tongren Medical Information System. The Tongren system contained information on the primary diagnosis and up to 16 other diagnoses for each hospitalization. Primary diagnosis in our analysis was defined as the diagnosis most associated with the chief complaint and was grouped into three mutually exclusive categories: AIDS-defining events (ADEs), non-AIDS-defining events (nADEs), and other causes. The ADEs category included all opportunistic illnesses listed in the 1993 CDC AIDS case definition (National Center for Infectious Diseases Division of HIV/AIDS). Recurrent bacterial pneumonia was defined as any bacterial pneumonia admission happened within >30 but ≤ 365 days of a previous such admission. The nADEs were defined as follows: cardiovascular disease (coronary heart diseases, cerebrovascular diseases, peripheral arterial diseases, primary pulmonary hypertension, and congestive heart failure), non-AIDS-defining cancer, hepatic disease (hepatic insufficiency, cirrhosis, digestive hemorrhages caused by esophageal varices, hepatic encephalopathy, hepatic transplantation, and hepatocellular carcinoma), metabolic disorder (diabetes mellitus and lactic acidosis), renal disease (acute/chronic renal failure, renal tubulopathy, nephrotic syndrome, hemodialysis or peritoneal dialysis commencement, and renal transplant), and skeletal disease (non-traumatic vertebral and non-vertebral fractures, and aseptic necrosis of the bone). The categorization was carried out by two blinded investigators independently. Annual hospitalization rates were calculated as the number of hospitalizations divided by 100 person-years at risk in each calendar year.

### Statistical analysis

We plotted crude hospitalization rates for all causes and the three diagnostic categories. Rates standardized to the 2014 distribution of variables that changed substantially over the study period were calculated. Poisson regression models were used to estimate unadjusted and adjusted incidence rate ratios (IRRs) and 95% confidence intervals (95% CI) of hospitalization rates, with generalized estimating equations with an independent correlation matrix to account for patients contributing more than one hospitalization. Laboratory value for hospitalization outcome analyzes was the first measurement made during their hospital admission or the closest measurement made 6 months prior to hospitalization. Statistical analyzes were conducted using Stata 15.0 (StataCorp LP, College Station, TX) and GraphPad 8 (GraphPad Software, La Jolla, CA, USA). A *p* < 0.05 was considered statistically significant.

## Results

### Characteristics of study sample

**Figure 1:**
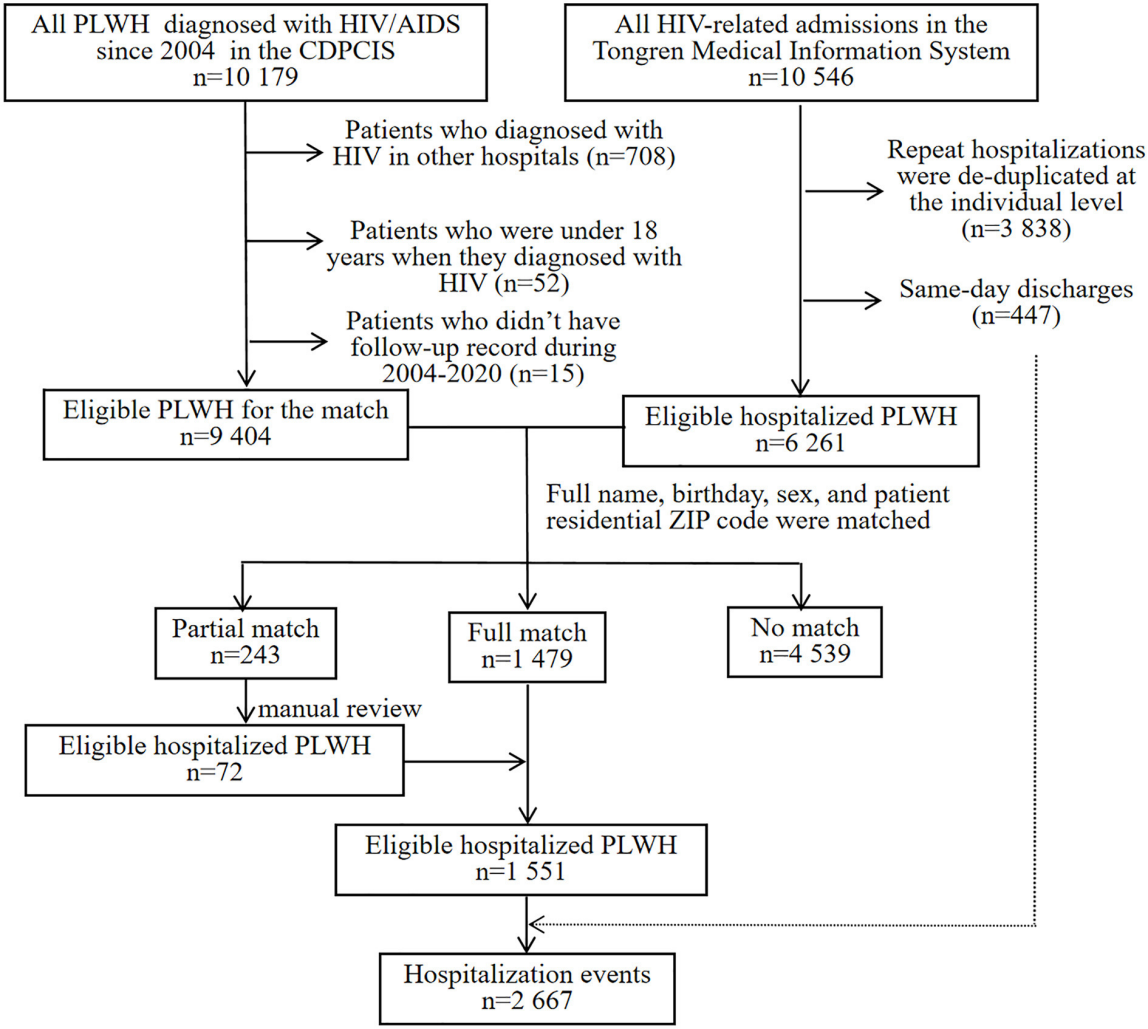
CDPCIS data and Tongren Medical Information System data set match process. HIV, human immunodeficiency virus; AIDS, acquired immune deficiency syndrome; PLWH, people living with HIV; CDPCIS, China Disease Prevention and Control Information System.

**Figure 2:**
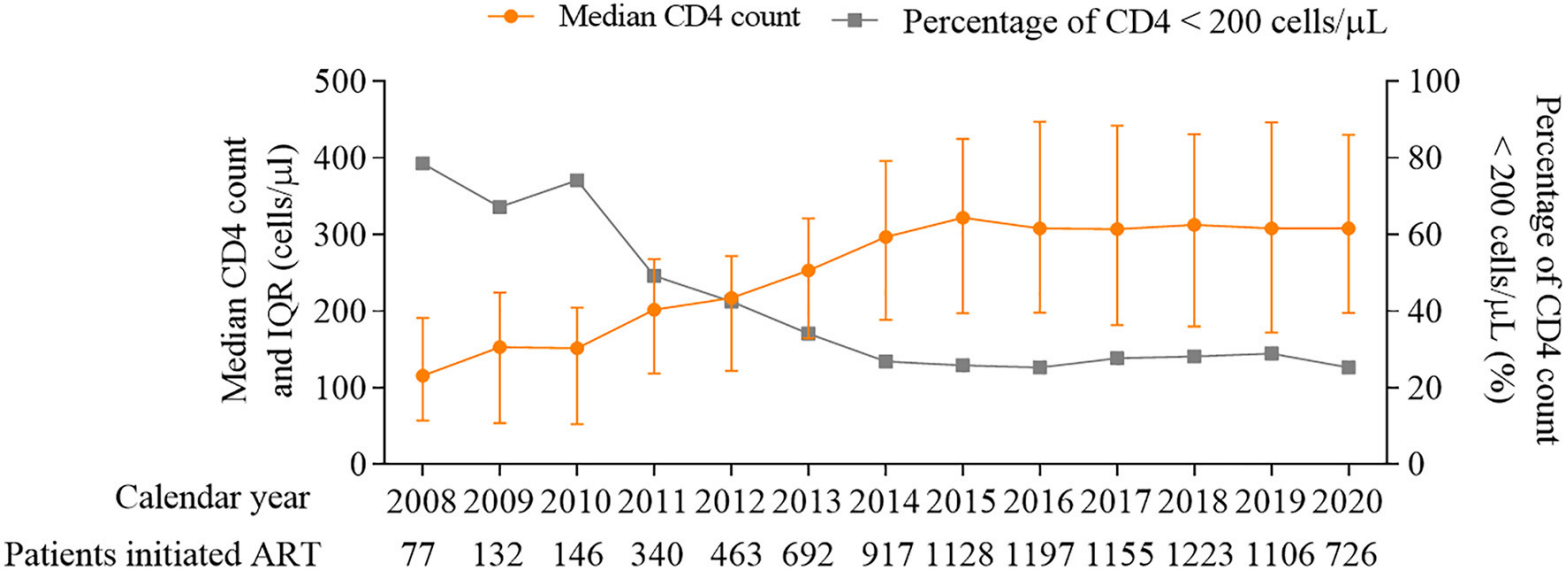
Median CD4 count (cells/μL) and percentage of patients with CD4 count < 200 cells/μL at ART initiation, 2008–2020. ART, antiretroviral therapy.

The matched data set included 10,179 PLWH and 10,546 hospitalization events. The final analytic sample after exclusions included 9,404 PLWH, among which 16.5% (1,551/9,404) were admitted to hospital at least one time (Figure 1). The cohort composed of 8,906 (94.7%) male and 498 (5.3%) female patients, with a median age of 29 [interquartile range (IQR) 25–37] years and a median CD4 count of 284 (IQR, 166–401) cells/μL at enrollment (Table 1). Over the study period, the number of patients initiated ART increased from 77 in 2008 to 726 in 2020 (Figure 2), the CD4 count at ART initiation increased from 116 (IQR, 57–191) cells/μL in 2008 to 322 (IQR, 197–425) cells/μL in 2015, and the percentage of patients with a CD4 count < 200 cells/μL decreased from 78.6% in 2008 to 25.8% in 2015 but did not further change significantly thereafter (Figure 2). The patients contributed a median of 2.9 years of follow-up (IQR, 0.3–5.6 years) and a total of 49,419 person-years. From 2008 to 2020, a mild increase in the median age of patients in care was observed [33 years (IQR, 28–39 years) to 34 years (IQR, 29–42 years, Table 1)]. The proportion of male patients increased from 80.7 to 94.8%, and the proportion of MSM increased from 57.1 to 79.2%. The first available CD4 count of patients in care in every calendar year increased from 283 (IQR, 181–395) cells/μL in 2008 to 570 (IQR, 416–742) cells/μL in 2020, and the proportion of PLWH with an HIV viral load (VL) ≤ 50 copies/mL increased from 41.5% in 2008 to 92.4% in 2020.

**Table 1:** Characteristics of HIV-infected patients at enrollment and across calendar years.

	**All patients at enrollment (*n* = 9,404)**	**Patients in care in 2008 (*n* = 410)**	**Patients in care in 2014 (*n* = 3,538)**	**Patients in care in 2020 (*n* = 8,349)**	***P*-value^a^**
Age, years^b^					
Median (IQR)	29 (25–37)	33 (28–39)	31 (26–39)	34 (29–42)	< 0.01
18–34	6,573 (69.9%)	239 (58.3%)	2,230 (63%)	4,413 (52.9%)	
35–49	2,181 (23.2%)	152 (37.1%)	1,048 (29.6%)	2,993 (35.8%)	
≥50	649 (6.9%)	19 (4.6%)	260 (7.3%)	943 (11.3%)	
Sex, *n* (%)					<0.01
Male	8,906 (94.7%)	331 (80.7%)	3,298 (93.2%)	7,920 (94.8%)	
Female	498 (5.3%)	79 (19.3%)	240 (6.8%)	429 (5.2%)	
HIV acquisition risk factors, *n* (%)					<0.01
MSM	7,419 (78.9%)	234 (57.1%)	2,784 (76.7%)	6,615 (79.2%)	
Heterosexual	1,176 (12.5%)	76 (18.5%)	378 (10.7%)	1,048 (12.6%)	
Other routes	809 (8.6%)	100 (24.4%)	376 (10.6%)	686 (8.2%)	
CD4 count, cells/μL, *n* (%)^c^					
Median (IQR)	284 (166–401)	283 (181–395)	451 (338–584)	570 (416–742)	<0.01
VL ≤ 50 copies/mL, *n* (%)^d^	152 (1.7%)	170 (41.5%)	3,069 (86.7%)	7,714 (92.4%)	<0.01

HIV, human immunodeficiency virus; IQR, interquartile range; MSM, men who have sex with men; VL, viral load.

a *P*-values compare calendar years 2008, 2014, and 2020, estimated using χ^2^ tests for categorical variables and Kruskal–Wallis tests for continuous variables.

b Age was assessed on 1 January of each calendar year.

c CD4 count at enrollment was the closest measurement a month before to a month after the diagnostic date and was missing for 498 patients. The time-updated CD4 count was the first available measurements for each calendar year, or, if unavailable, in the last 6 months of the previous year or first 6 months of the following year. Time-updated CD4 count was missing for 2471 (5.0%) person-years.

d VL at enrollment was the closest measurement a month before to a month after the diagnostic date and was missing for 525 patients. Time-updated VLs were the first available measurements for each calendar year, or, if unavailable, in the last 6 months of the previous year or first 6 months of the following year. Time-updated VL was missing for 2,866 (5.8%) person-years.

### Characteristics for hospitalizations

During the observed person-time, 1,551 PLWH were hospitalized at least one time, with a total of 2,667 hospitalization events. Among them, 91.7% were male patients (Table 2). The awareness rate of HIV infection was 66.4% in hospitalized PLWH and 47.1% in ART-naive individuals (data not shown). MSM is the main risk factor for HIV transmission among hospitalized patients (62.8%). The median age at admission was 35 (IQR, 29–46) years. Most admissions were seen in the outpatient department (81.6%). Overall, the inpatient mortality was 2.8% (44/1,551), among which 66.0% (29/44) happened in the first 30 days after admission. The rate of ICU admission was 1.4% (38/2,667). ADEs were the most common cause of death and ICU admission (72.7 and 78.9%, respectively). The median hospital length of stay was 15 (IQR, 8–26) days. The most common reasons for hospitalizations were ADEs in 60.4% of hospitalization events, and the three most common diagnoses in ADEs were pneumocystis pneumonia (37.8%), tuberculosis (33.9%), and cytomegalovirus infection (12.8%, Table 3). For nADE hospitalizations (11.4%), the three most common diagnoses were non-AIDS-defining cancer (29.7%), hepatic disease (28.7%), and cardiovascular disease (23.8%). For hospitalizations for other causes (28.2%), non-AIDS-defining infections (44.4%) were the most common diagnosis.

**Table 2:** Demographic and clinical characteristics, overall hospitalization, and ART status of admitted HIV-infected patients in 2008–2020.

		**Hospitalized late presenters**		**Hospitalized ART-experienced patients**
	**All-cause hospitalization (*n* = 2,667)**	**ART-naive (*n* = 1,161)**	**ART <6 mo (*n* = 698)**	**ART ≥6 mo (*n* = 808)**
Age at admission, years				
Median (IQR)	35 (29–46)	31 (28–45)	29 (28–46)	39 (31–51)
18–34	1,272 (47.7%)	605 (52.1%)	383 (54.9%)	284 (35.1%)
35–49	903 (33.9%)	392 (33.8%)	200 (28.7%)	311 (38.5%)
≥50	492 (18.4%)	164 (14.1%)	115 (16.5%)	213 (26.4%)
Sex, *n* (%)				
Male	2,445 (91.7%)	1,071 (92.2%)	641 (91.8%)	733 (90.7%)
Female	222 (8.3%)	90 (7.8%)	57 (8.2%)	75 (9.3%)
Type of admission, *n* (%)				
Emergency	490 (18.4%)	231 (19.9%)	92 (13.2%)	167 (20.9%)
Outpatient	2,177 (81.6%)	930 (80.1%)	606 (86.8%)	641 (79.1%)
HIV acquisition risk factors, *n* (%)				
MSM	1,675 (62.8%)	740 (63.7%)	448 (64.2%)	487 (60.3%)
Heterosexual	470 (17.6%)	200 (17.2%)	119 (17.0%)	151 (18.7%)
Other routes	522 (19.6%)	221 (19.0%)	131 (18.8%)	170 (21.0%)
CD4 count at admission, cells/μL^a^				
Median (IQR)	108 (29–289)	44 (14–161)	112 (44–229)	280 (127–461)
≤ 200	1,489 (55.8%)	816 (70.3%)	422 (60.5%)	251 (31.1%)
VL ≤ 50 copies/mL at admission, *n* (%)^b^	783 (29.4%)	8 (0.7%)	171 (24.5%)	604 (74.8%)
Primary diagnosis				
ADE	1,611 (60.4%)	863 (74.3%)	461 (66.0%)	287 (35.5%)
nADE	303 (11.4%)	66 (5.7%)	68 (9.7%)	169 (20.9%)
Others	753 (28.2%)	232 (20%)	169 (24.3%)	352 (43.6%)
ART regimen at admission, *n* (%)				
NNRTI-based	1,426 (95.3%)	-	659 (94.8%)	767 (95.6%)
PI-based	59 (3.9%)	-	30 (4.3%)	29 (3.6%)
INI-based	12 (0.8%)	-	6 (0.9%)	6 (0.7%)
Cost of hospitalization, CNY, Median (IQR)	21 896 (10,857–40,528)	31 700 (18,147–49707)	17 265 (9,188–34,92)	8 027 (14,347–27,863)
Length of hospitalization stay, days,				
Median (IQR)	15 (8–26)	22 (14–32)	13 (7–22)	10 (6–17)
ICU admission rate, *n* (%)	38 (1.4%)	27 (2.3%)	2 (0.3%)	9 (1.1%)

ART, antiretroviral therapy; HIV, human immunodeficiency virus; MSM, men who have sex with men; IQR, interquartile range; VL, viral load; ADEs, AIDS-defining events; nADEs, non-AIDS-defining events; NNRTI, non-nucleoside reverse transcriptase inhibitor; PI, protease inhibitor; INI, integrase inhibitor; CNY, China yuan.

a CD4 count was the first measurement made during patients' hospital admission or the closest measurement in the 6 months prior to hospitalization. CD4 count at admission was missing for 394 hospitalizations.

b VL was the first measurement made during their hospital admission or the closest measurement in the 6 months prior to hospitalization. VL at admission was missing for 624 hospitalizations.

**Table 3:** Distribution of discharge diagnoses for 2,667 hospitalizations among 9,404 patients.

**No. (%)**	**Diagnosis**
**ADEs (*****n*** **=** **1,611, 60.4%)**^**a**^	
621 (37.8%)	*Pneumocystis* pneumonia
505 (33.9%)	Tuberculosis
206 (12.8%)	Cytomegalovirus infection
192 (11.9%)	Candidiasis
156 (9.7%)	Recurrent bacterial pneumonia
98 (6.1%)	Cryptococcosis
55 (3.4%)	Kaposi sarcoma
52 (3.2%)	Lymphoma
50 (3.1%)	Varicella-Zoster virus
27 (2.7%)	Toxoplasma gondii encephalitis
**nADEs (*****n*** **=** **303, 11.4%)**	
90 (29.7%)	Non-AIDS-defining cancer
87 (28.7%)	Hepatic disease
72 (23.8%)	Cardiovascular disease
30 (9.9%)	Metabolic disorder
15 (5.0%)	Renal disease
9 (3.0%)	Skeletal disease
**Other causes (*****n*** **=** **753, 28.2%)**^**b**^	
334 (44.4%)	Non AIDS-defining infection
59 (7.8%)	Pregnancy and newborn
32 (4.2%)	Hemocytopenia
29 (3.9%)	Injury
20 (2.7%)	Benign tumors

ADEs, AIDS-defining events; nADEs, non-AIDS-defining events.

a Total percentage exceeds 100% because some patients had > 1 AIDS-defining illnesses.

b Five most frequent diagnostic categories.

Among all hospitalized patients, 69.7% were late presenters, defined as ART-naive individuals and those who had only recently initiated treatment (<6 months on ART). These patients are more likely to be hospitalized for ADEs than those on ART for ≥ 6 months (defined as ART-experienced PLWH). A significantly lower median CD4 count (ART-naive vs. ART <6 months vs. ART >6 months: 44 cells/μL vs. 112 cells/μL vs.280 cells/μL, Table 2) and more detectable VL at admission (99.3% vs. 65.5% vs. 25.2%) were found among late presenters. Furthermore, late presenters tended to have greater disease burden, with a higher inpatient cost (31 700 CNY vs. 17 265 CNY vs. 8 027 CNY) and a longer length of hospitalization stay (22 days vs.13 days. vs.10 days).

### Time trends for hospitalization rates and proportion of hospitalization causes

**Figure 3:**
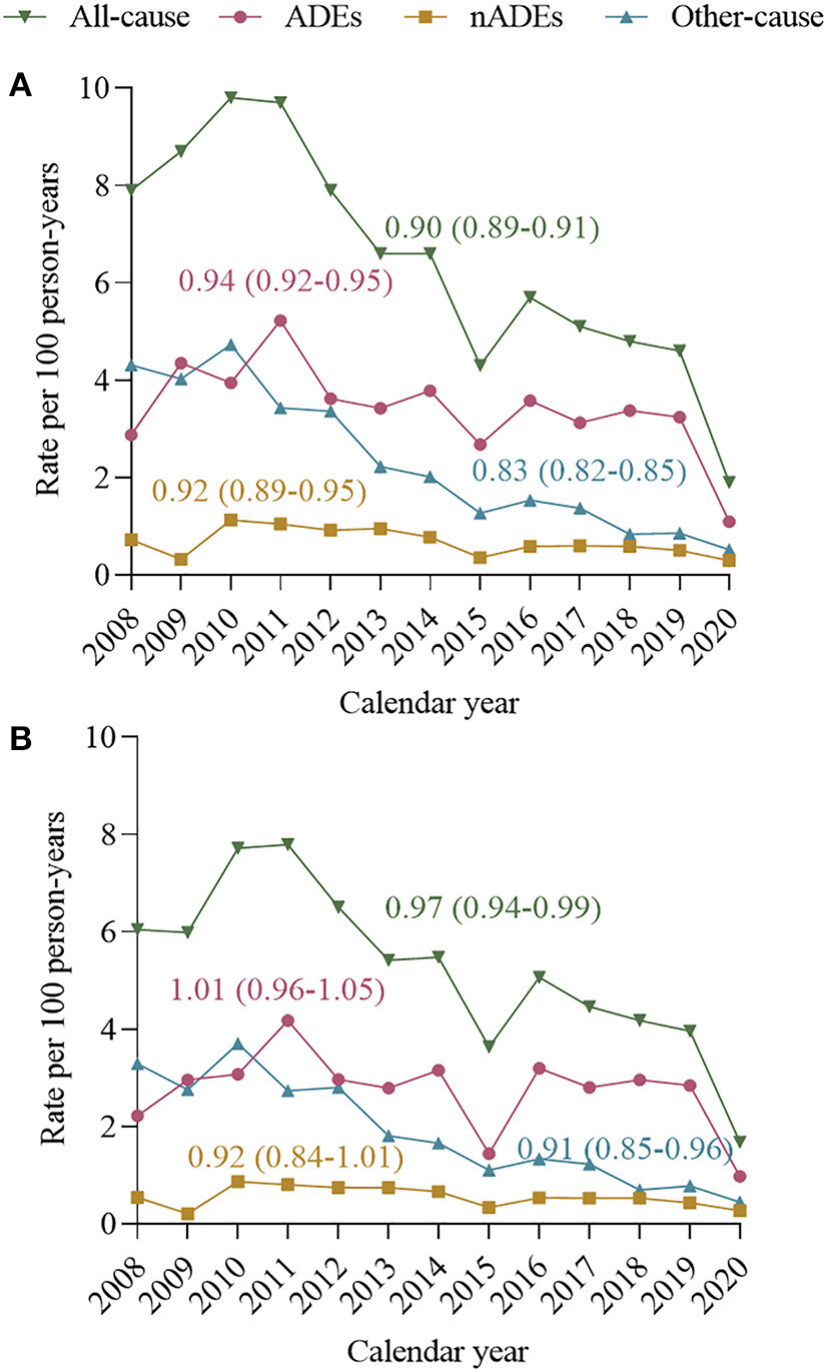
Incidence rate ratios (IRRs) and 95% confidence intervals (95% CI) calculated using Poisson regression models. Annual hospitalization rates and mean rate change, 2008–2020, for all-cause hospitalizations, ADE-related hospitalization nADE-related hospitalization and other-causes hospitalization. **(A)** Unadjusted annual all-cause and cause-specific hospitalization rates. **(B)** Annual all-cause and cause-specific hospitalization rates, standardized to age, sex, HIV transmission risk, CD4 count, and VL distribution of person-years in 2014. Standardization strata were defined according to the following categories: age 18–34, 35–49, and ≥50 years; CD4 count ≤ 200 and >200 cells/μL; viral load ≤ 50 and >50 copies/mL. ADE, AIDS-defining events; nADEs, non-AIDS-defining events.

Unadjusted all-cause hospitalization rates decreased from 7.9 per 100 person-years in 2008 to 1.9 in 2020, with a mean change of −10% per year [IRR per year 0.90 (0.89–0.91), Figure 3A]. The decline in unadjusted hospitalization rates was observed over the study period for ADEs [IRR per year 0.94 (0.92–0.95)], nADEs [IRR per year 0.92 (0.89–0.95)], and other causes [IRR per year 0.83 (0.82–0.85)]. After standardizing of age, sex, HIV transmission risk factor, CD4 count, and VL distribution in 2014 (Figure 3B), we observed decreases in hospitalization rates for all-cause [IRR per year 0.97 (0.94–0.99)] and other-cause hospitalizations [IRR per year 0.91 (0.85–0.96)], but hospitalization rates of ADE-related [IRR per year 1.01 (0.96–1.05)] and nADE-related hospitalizations [IRR per year 0.92 (0.84–1.01)] appeared stable.

**Figure 4:**
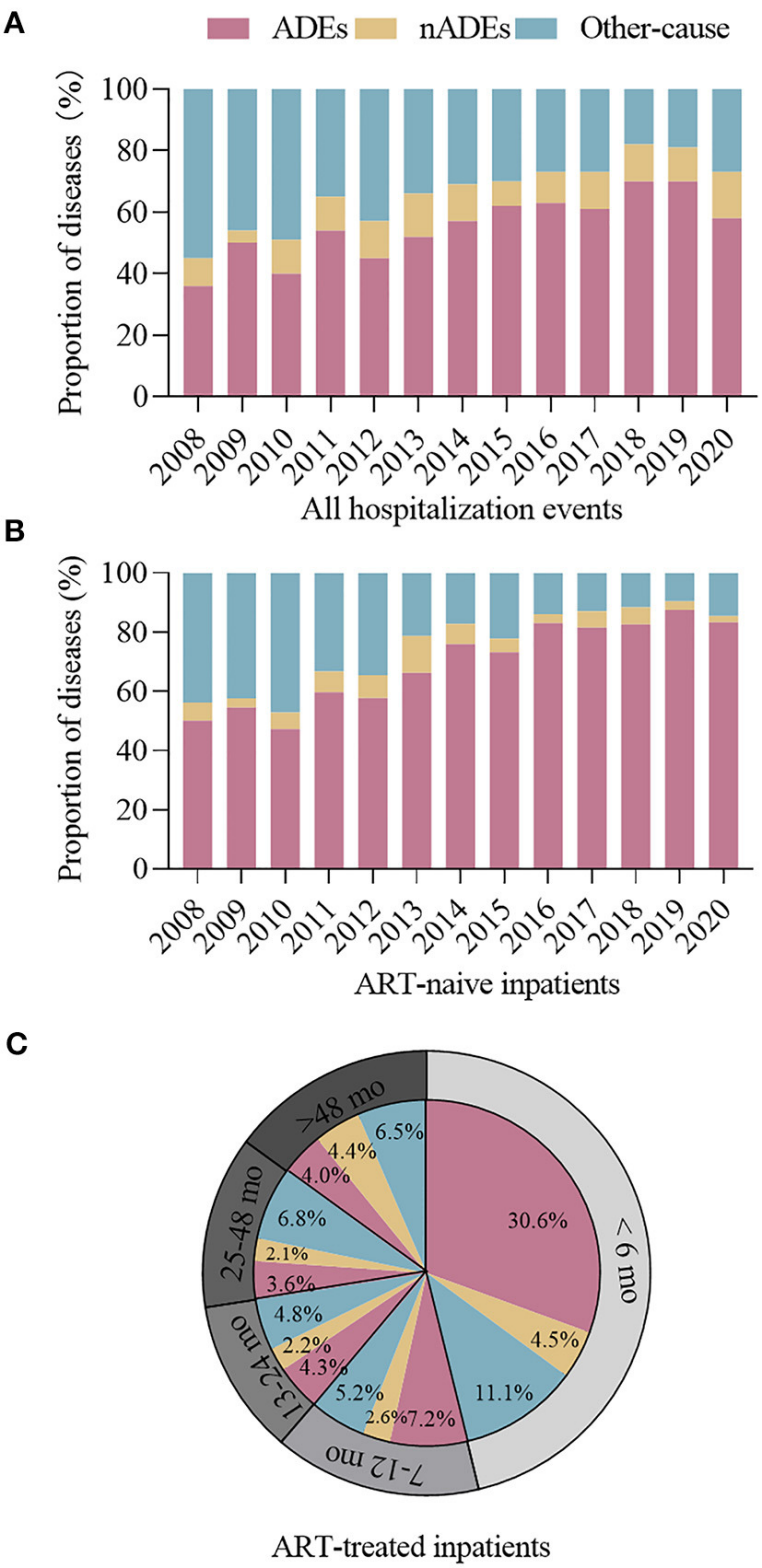
Proportion of hospitalizations for ADEs, nADEs, and other causes among **(A)** all hospitalized cases and **(B)** ART-naive cases, 2008–2020. **(C)** Proportion of hospitalizations for ADEs, nADEs, and other causes among cases with different ART durations. ADE, AIDS-defining events; nADEs, non-AIDS-defining events; ART, antiretroviral therapy.

**Figure 5:**
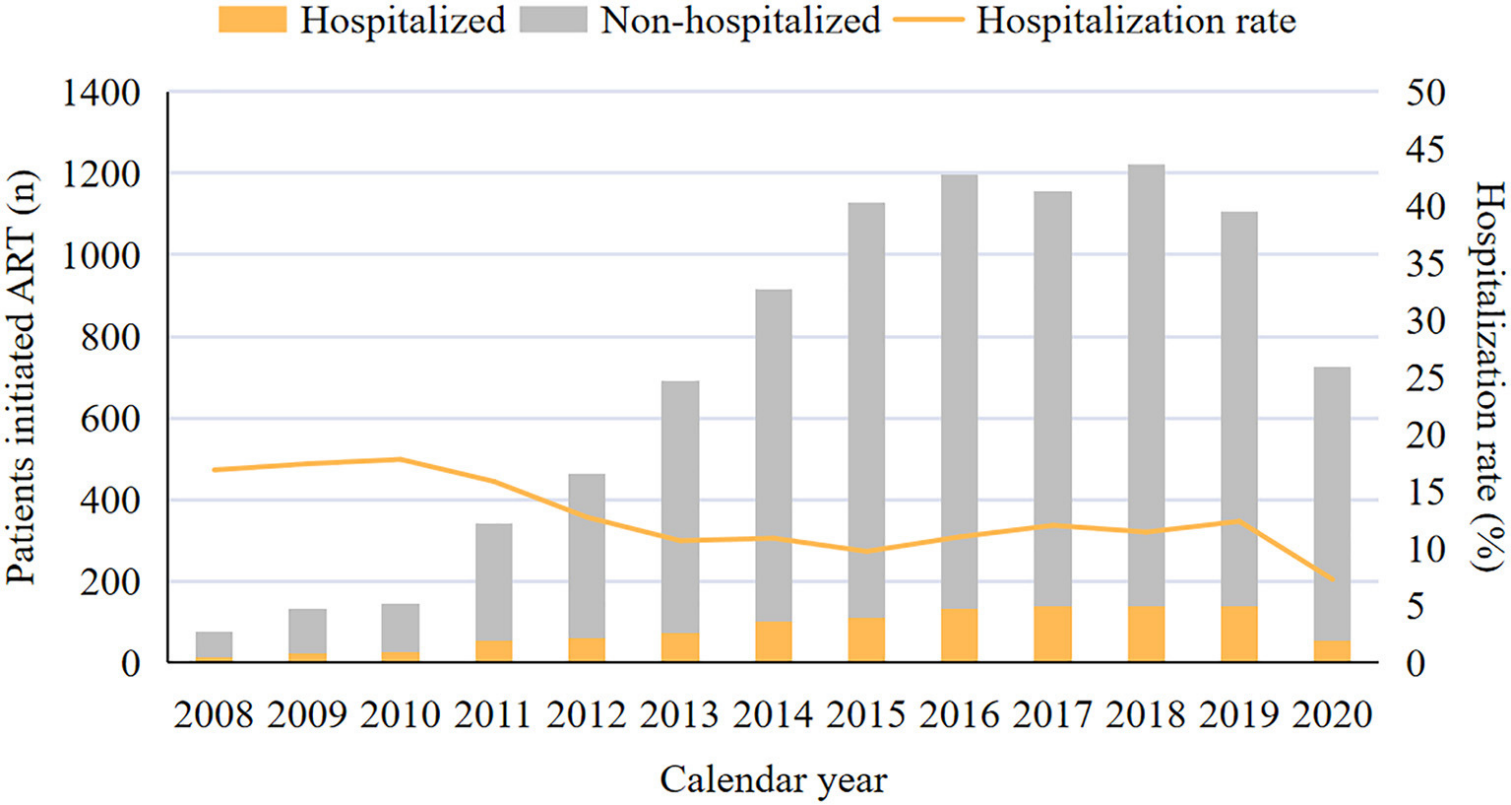
Proportion of hospitalized patients at ART initiation, 2008–2020. ART, antiretroviral therapy.

The changes in the proportion of hospitalization causes are shown in Figure 4. Overall, the proportion of ADE-related hospitalizations increased from 36.3% in 2008 to 57.4% in 2020, and that of nADE-related hospitalizations slightly increased but remained low (9.0% in 2008 to 15.4% in 2020, Figure 4A). In ART-naive inpatients, the proportion of ADE-related hospitalizations increased from 43.8% in 2008 to 83.3% in 2020 (Figure 4B). However, the proportion of hospitalized patients to those who initiated ART declined from 16.9% in 2008 to 7.3% in 2020 (Figure 5). For patients who were treated with ART, hospitalization was highest during the first 6 months after ART (46.2%, Figure 4C), when ADEs were still the leading causes of hospitalizations (30.6%).

### Risk factors for hospitalizations

In multivariable models, patients with an older age (35–49 years vs. ≤ 34 years, IRR for all-cause, ADE-related, and nADE-related hospitalizations: 1.38, 1.38, and 2.55, respectively; ≥50 years vs. ≤ 34 years, IRR: 2.10, 2.00, and 5.05, respectively, Table 4) and those infected by heterosexual transmission (heterosexual vs. MSM, IRR: 1.56, 1.53, and 1.46, respectively) or other routes (heterosexual vs. MSM, IRR: 1.97, 1.81, and 2.66, respectively) had a higher risk of all-cause, ADEs-related, and nADEs-related hospitalizations. On the contrary, ART > 6 months (IRR: 0.21, 0.11, and 0.49, respectively) and diagnosed between July 2014 and December 2020 (vs. January 2008–June 2014, IRR 0.57, 0.73, 0.55, respectively) were associated with a lower risk of all-cause, ADEs-related, and nADEs-related hospitalizations. Patients with a CD4 count ≤ 200 cells/μL (vs. CD4 > 200 cells/μL, IRR for all-cause and ADE-related hospitalizations: 3.95 and 7.45, respectively) or a VL > 50 copies/mL (vs. VL ≤ 50 copies/mL, IRR: 1.45 and 1.64, respectively) had a higher risk of all-cause and ADE-related hospitalizations. Female patients had a lower risk of hospitalizations due to ADEs than male patients (IRR: 0.55).

**Table 4:** Multivariable analyzes of factors associated with hospitalization for all causes, ADEs, and nADEs.

	**IRR (95% CI)** ^**a**^		
	**All-cause hospitalizations (*n* = 2,667)**	**ADEs-related hospitalizations (*n* = 1,611)**	**nADEs-related hospitalizations (*n* = 303)**
Age at admission, years			
18–34	1.00 (Ref)	1.00 (Ref)	1.00 (Ref)
35–49	**1.38 (1.26–1.51)**	**1.38 (1.23–1.55)**	**2.55 (1.94–3.37)**
≥50	**2.10 (1.88–2.35)**	**2.00 (1.73–2.32)**	**5.05 (3.68–6.91)**
Sex			
Male	1.00 (Ref)	1.00 (Ref)	1.00 (Ref)
Female	0.91 (0.78–1.06)	**0.55 (0.43–0.70)**	0.83 (0.55–1.26)
HIV acquisition risk factors			
MSM	1.00 (Ref)	1.00 (Ref)	1.00 (Ref)
Heterosexual	**1.56 (1.39–1.75)**	**1.53 (1.32–1.77)**	**1.46 (1.04–2.07)**
Other routes	**1.97 (1.77–2.20)**	**1.81 (1.57–2.09)**	**2.66 (2.00–3.55)**
ART use, *n* (%)			
Late presenters	1.00 (Ref)	1.00 (Ref)	1.00 (Ref)
ART experienced	**0.21 (0.19–0.23)**	**0.11 (0.10–0.13)**	**0.49 (0.38–0.62)**
CD4 count at admission, cells/μL^b^			
>200	1.00 (Ref)	1.00 (Ref)	1.00 (Ref)
≤ 200	**3.95 (3.34–4.67)**	**7.45 (5.92–9.36)**	1.08 (0.58–2.03)
Missing	**1.31 (1.14–1.49)**	**1.94 (1.58–2.38)**	0.89 (0.64–1.23)
VL at admission, copies/mL			
≤ 50	1.00 (Ref)	1.00 (Ref)	1.00 (Ref)
>50	**1.45 (1.10–1.90)**	**1.64 (1.18–2.29)**	0.24 (0.03–1.75)
Missing	0.93 (0.82–1.06)	1.04 (0.87–1.24)	**0.68 (0.49–0.96)**
Calendar period			
June, 2014	1.00 (Ref)	1.00 (Ref)	1.00 (Ref)
July, 2014–2020	**0.57 (0.52–0.61)**	**0.73 (0.65–0.81)**	**0.55 (0.44–0.70)**

ADE, AIDS-defining events; nADE, non-AIDS-defining events; IRR, incidence rate ratio; CI, confidence interval; ref, reference; HIV, human immunodeficiency virus; MSM, men who have sex with men; VL, viral load.

a IRRs, 95% CIs, and *P*-values were obtained from Poisson regression models. Results in bold are statistically significant (*P* < 0.05).

b CD4 count and VL used in this analysis were the first available measurements for each calendar year, or, if unavailable, in the last 6 months of the previous year or first 6 months of the following year.

## Discussion

With evidence of the benefits of ART, China launched the National HIV/AIDS Response Program in 2003, yet the number of newly diagnosed cases and the overall mortality have remained high (2, 13). According to the National Center for AIDS/STD Prevention and Control, there were 1,053,000 PLWH and 351,000 accumulated death cases in China in 2020. Moreover, a late HIV diagnosis is a serious public health problem. A study indicated that the rate of advanced HIV disease in China ranged from 35.5 to 42.1% from 2010 to 2014 (14). In this record-based retrospective cohort study, a continually growing number of newly diagnosed cases and younger cases was identified from 2008 to 2020, which was mainly attributed to expanding HIV-testing coverage and enlarging MSM population (15). The prevalence of late HIV diagnosis decreased below 30% after 2014, which is lower than that in other regions of China (16, 17). Improvements in immunologic status were observed not only in patients in HIV care but also at the initiation of ART. In addition, the proportion of hospitalized patients to those who initiated ART decreased. However, these improvements were much less pronounced after 2014–2015. These data suggest that regardless of advancements made in fighting the HIV epidemic, there are still many challenges before definitely putting an end to it.

Colasanti et al. underlined the importance of monitoring hospitalizations among persons with HIV (18). They considered every preventable hospitalization as a failure of public health. Studies conducted in high-income countries reported a declining rate of hospitalization in PLWH since ART became widely available; the major decline was detected for ADEs (4, 19, 20). We showed a decline in the overall hospitalization rate and the risk of hospitalizations over 13 years (2008–2020). However, when considering different reasons for hospitalizations, we noted that ADEs were the most common reason for hospitalizations, which is consistent with data from other countries (21, 22). Moreover, although the unadjusted rate of ADE-related hospitalizations declined from 2.87 per 100 person-years in 2008 to 1.09 per 100 person-years in 2020, the decline was no longer present after adjusting for the variables that changed significantly. This phenomenon could be explained by the fact that 70% of the hospitalized cases in our cohort were late presenters. Most of them were extremely immunosuppressed and had already progressed to AIDS, suggesting that late treatment remains a serious problem. Furthermore, the low CD4 cell count at HIV diagnosis indicates that late treatment initiation was mainly due to late diagnosis. Studies estimated that newly diagnosed patients in China had been infected with HIV for a mean of 6.3 years at the time of diagnosis, and the time from HIV diagnosis to starting ART was 6 months (23, 24), which shows much room for improvement in initiating treatment earlier.

Multiple countries have gradually advanced the timing of ART initiation and expanding HIV testing services (25). In 2015, World Health Organization (WHO) recommended that all people diagnosed with HIV should start ART at any CD4^+^ T-cell count. However, different countries have been faster or slower in translation of WHO guidance into national policies and into clinical practice at the service delivery level (26). Furthermore, the process implementation from screening to treatment remains incomplete, and PLWH often fails to initiate treatment in time (27). In our cohort, the proportion of ADE-related hospitalizations increased over time, especially in ART-naive inpatients, suggesting an urgent need to intensify efforts to find and link HIV care. With the extension of ART duration, a decrease in the proportion of hospitalizations, especially for ADEs, was observed, indicating the protective effect of ART against hospitalizations. Possible reasons for an early hospitalization risk after ART initiation include ART toxicity, immune reconstitution inflammatory syndrome (IRIS), or time required for ART to become effective. We did not directly assess the contribution of IRIS because these clinical diagnoses were not systematically recorded; still, we noted that among patients treated with ART for <6 months, about two-thirds were hospitalized for ADEs. Therefore, patients at high risk for the development of IRIS should be identified, and the risk of IRIS should be weighed in when deciding the timing of ART initiation (28).

As a result of the prolonged lifespan in the ART era, there is an increasing focus on non-AIDS events (29–31). A study on a European population of HIV-infected patients from 2001 to 2009 reported considerable mortality associated with non-AIDS events, exceeding that of AIDS events. In high-income countries, nADEs have become the most common reason for hospitalization, and the rates for nADE-related hospitalizations have remained stable or increased over the past two decades (32–35). Our adjusted analysis showed that the nADE-related hospitalization rates were low and stable and mainly happened in ART-experienced patients. This relative lack of nADE-associated hospitalizations may be related to the relatively young age of patients in our cohort (32, 36, 37) and suggests that HIV-induced immunodeficiency and viremia independently participate in the increasing risk of nADEs (29). Still, we noted that the percentage of patients older than 50 years in our cohort increased from 4.6% in 2008 to 11.3% in 2020. As the older adult population increases and the ART is prolonged, the concept of monitoring nADEs must become more important (38).

Markers indicating severe disease, including older age, late presenters, CD4 counts ≤ 200 cells/μL, and VLs > 50 copies/mL, were associated with a higher risk of hospitalizations. MSM had a significantly lower risk of hospitalization than non-MSM-infected patients, which probably results from the perceived increased risk of HIV transmission in MSM and increased coverage of HIV testing among MSM. Most of the enrolled HIV-positive MSM are probably in the early stages of the disease progression (2). It was reported that compared with those infected through other routes in China, HIV-positive MSM patients are younger, better educated, and highly adhere to the provided care (39). Findings regarding the association between gender and hospitalization were mixed. Studies conducted in the United States reported a higher hospitalization rate among female PLWH, even after accounting for pregnancy-related hospitalizations (4, 5, 40). We found an association between male sex and increased risk of ADE-related hospitalizations, and the results were consistent with the findings of the study by Wang et al., which presented the pattern of HIV/AIDS-related deaths in China over the period 2000–2012 and reported increased AIDS-related mortality in male than in female patients (41).

The present study has several limitations. A main limitation of our study is that as we did not have access to hospitalization records from other hospitals, our findings are representative of a single center in China, and thus, results may not be generalizable to other settings. In addition, to avoid underestimation of hospitalization rates, a more stringent definition of LTFU was employed, which led to a relatively short median follow-up period over the course of 13 years. Second, instead of taking the first non-HIV diagnosis code, an approach to determine hospitalization causes among PLWH, we assigned the primary diagnosis as the one most associated with the chief complaint. Given concerns about the accuracy of diagnosis at the beginning of our study period, physician review may be less biased than traditional methods. Third, data extraction was hospitalization-driven. Due to the differences in the follow-up time and speed of cohort entry, the risk of hospitalization was different in patients. Therefore, data for patients not requiring hospitalization might be underestimated. Fourth, we calculated rates based on broad categories of causes of hospitalization to maintain statistical power. The trends and outcomes of specific diseases were not explored. Finally, data on other covariates (such as socioeconomic status, smoking habit, drug use, and mental health disorders) were not included; these factors could be associated with hospitalizations. Future studies should focus on them.

## Conclusion

Despite the impressive investments and advancements against HIV over the past decades in China, ADEs are still the most serious problem. Non-MSM, older patients, late presenters, and immune-depleted individuals are at greater risk of hospitalization. We emphasize the need for continued efforts to diagnose and treat HIV earlier to avoid deteriorating to the stage of needing hospitalization.

## Data availability statement

The original contributions presented in the study are included in the article/Supplementary files, further inquiries can be directed to the corresponding authors.

## Ethics statement

The studies involving human participants were reviewed and approved by Human Science Ethical Committee of Beijing Ditan Hospital, Capital Medical University. Written informed consent for participation was not required for this study in accordance with the national legislation and the institutional requirements.

## Author contributions

YL, HZ, and JH conceived and designed the study. YL, YH, JX, LW, and HL conducted the data analysis, literature review, and drafted the manuscript and assisted with data management. YL, YH, JX, JH, and HZ involved in the study supervision, data collection, and interpretation of the data. All authors contributed to the revision of the manuscript and approved the final version.

## Funding

This research was funded by the Beijing Municipal Administration of Hospitals Clinical Medicine Development of Special Funding Support (ZYLX202126), Beijing Municipal Science and Technology Commission (Z191100006619045), and Beijing Municipal Administration of Hospitals' Ascent Plan (DFL20191802).

## Conflict of interest

The authors declare that the research was conducted in the absence of any commercial or financial relationships that could be construed as a potential conflict of interest.

## Publisher's note

All claims expressed in this article are solely those of the authors and do not necessarily represent those of their affiliated organizations, or those of the publisher, the editors and the reviewers. Any product that may be evaluated in this article, or claim that may be made by its manufacturer, is not guaranteed or endorsed by the publisher.
