# Supplementary material for: Trends in rates and causes of hospitalization among people living with HIV in the antiretroviral therapy era: A retrospective cohort study in China, 2008–2020
Source: Front Public Health. 2022 Nov 8;10:1000942. doi: 10.3389/fpubh.2022.1000942 (PMC9680952; doi:10.3389/fpubh.2022.1000942)
Supplement: Supplementary file 1 [file Data_Sheet_1.docx]

https://www.jianguoyun.com/p/DY2oP60Qo47PChic4dcEIAA

https://www.jianguoyun.com/p/DelZrFsQo47PChih4dcEIAA

https:// www.jianguoyun.com/c/ sd/153c723/36d19bcb9ef39609
